# Financial sustainability of payment models for office-based opioid treatment in outpatient clinics

**DOI:** 10.1186/s13722-021-00253-7

**Published:** 2021-07-05

**Authors:** Dominic Hodgkin, Constance Horgan, Gavin Bart

**Affiliations:** 1 Institute for Behavioral Health, Heller School for Social Policy and Management, Brandeis University, Waltham, United States; 2 Department of Medicine, University of Minnesota Medical School and Division of Addiction Medicine, Hennepin Healthcare, Minneapolis, United States

**Keywords:** Office-based opioid treatment, Buprenorphine, Medication treatment of opioid use disorder, Reimbursement, Payment models, Financial sustainability, Medicaid

## Abstract

**Background:**

Office-Based Opioid Treatment (OBOT) is a delivery model which seeks to make medications for opioid use disorder (MOUD), particularly buprenorphine, widely available in general medical clinics and offices. Despite evidence supporting its effectiveness and cost-effectiveness, uptake of the OBOT model has been relatively slow. One important barrier to faster diffusion of OBOT may be the financial challenges facing clinics that could adopt it.

**Methods:**

We review key features and variants of the OBOT model, then discuss different approaches that have been used to fund it, and the findings from previous economic analyses of OBOT’s impact on organizational finances. We conclude by discussing the implications of these analyses for the financial sustainability of the OBOT delivery model.

**Results:**

Like other novel services, OBOT poses challenges for providers due to its reliance on services which are ‘non-billable’ in a fee-for-service environment. A variety of approaches exist for covering the non-billable costs, but which approaches are feasible depends on local payer policies. The scale of the challenges varies with clinic size, organizational affiliations and the policies of the state where the clinic operates. Small clinics in a purely fee-for-service environment may be particularly challenged in pursuing OBOT, given the need to fund a dedicated staff and extra administrative work. The current pandemic may pose both opportunities and challenges for the sustainability of OBOT, with expanded access to telemedicine, but also uncertainty about the durability of the expansion.

**Conclusion:**

The reimbursement environment for OBOT delivery varies widely around the US, and is evolving as Medicare (and possibly other payers) introduce alternative payment approaches. Clinics considering adoption of OBOT are well advised to thoroughly investigate these issues as they make their decision. In addition, payers will need to rethink how they pay for OBOT to make it sustainable.

## Introduction

Opioid use disorder (OUD) prevalence, poisonings, and deaths have increased substantially in the U.S. during the past decade. Nearly 2 million Americans currently have an OUD [1], and opioid overdoses accounted for over 50,000 deaths in 2019 [2]. Effective treatments for OUD exist, including medications approved by the US Food and Drug Administration (buprenorphine, methadone, and naltrexone), which are viewed as an essential component of evidence-based care [3, 4]. However, only about one in five individuals with an OUD receive any treatment [1, 5]. Among those who do, many do not receive medication for OUD (MOUD) [6–8].

A number of barriers contribute to low uptake of MOUD, including shortages of addiction treatment providers [9, 10], failure to recognize OUD due to inadequate clinician training or system fragmentation, insurance coverage limitations [11], stigma, and patient lack of readiness to address substance use issues [12–22]. In addition, for decades, in the US MOUD was largely only available as methadone, limited to specialized opioid treatment programs (OTPs). In the last two decades, Office-Based Opioid Treatment (OBOT) has emerged as a delivery model which seeks to make MOUD treatment, particularly with buprenorphine, widely available in general medical settings. There is evidence supporting the effectiveness of outpatient buprenorphine relative to no treatment [23–25]. However, uptake of the OBOT model has been relatively slow, with a small minority of physicians (under 6%) obtaining the federal “X” waiver required to prescribe buprenorphine [26], and low rates of prescribing among the few clinicians with waivers [27]. This may change with the US government’s recent decision to stop requiring clinicians to undergo special training in order to obtain a waiver [28].

One important barrier to faster diffusion of OBOT may be financial challenges facing the clinics that could adopt it. Prior research has found buprenorphine prescribing cost-effective from a societal perspective [29]. However, this finding does not imply cost-effectiveness from the clinic’s perspective, as a clinic must bear the additional costs of conducting OBOT without typically being rewarded for any resulting improvement in patient outcomes. This implies a lack of financial sustainability for individual provider organizations, which could be an important reason for the slow diffusion of OBOT. Numerous studies have reported that reimbursement is a challenge for clinics seeking to deliver OBOT services. Problems cited include that some of the services involved are not reimbursable, low reimbursement rates for other services, and caps on the number of patients per provider [31–32].

This paper therefore sets aside the societal perspective, and seeks instead to examine under what conditions OBOT is sustainable for individual clinics. We first review key features of the OBOT model, then discuss different approaches that have been used to fund it, including Medicare’s recent bundled payment initiative. We also review the findings from previous economic analyses of OBOT’s impact on organizational finances. We conclude by discussing the implications of these analyses for the financial sustainability of the OBOT delivery model. We recognize that nonfinancial factors too have important effects on sustainability (e.g. organizational culture, staff ideology), but defer their consideration to other studies.

## Key components of OBOT

In the US, for several decades up until 2000 the dominant form of MOUD was methadone, and federal law limited its dispensing to specialized “opioid treatment programs” (OTPs). This changed in 2000 when the Drug Addiction Treatment Act (DATA) allowed physicians to prescribe buprenorphine/naloxone in the context of primary care, provided they completed 8 h of training and received a Drug Enforcement Administration (DEA) waiver number—and, subsequently in 2016, 24 h for select non-physician advanced practice providers such as nurse practitioners and physician assistants. (Those training requirements were eliminated in April 2021). In the US, this approach is often referred to as Office-Based Opioid Treatment (OBOT), although more recently some proponents have expanded the term to “Office-Based Addiction Treatment” (OBAT), applying similar approaches to other substance use disorders. By June 2021, there were over 100,000 providers with DEA waivers [33]. OBOT is being offered in a variety of clinical settings, including federally qualified health centers (FQHCs), traditional primary care clinics, other integrated primary care clinics, Certified Community Behavioral Health Clinics (an emerging model), hospital-based clinics and opioid treatment programs (OTPs). This paper does not address OBOT delivery in OTPs given their very different regulatory setup.

We next describe 3 key dimensions of OBOT design issues that vary across programs. These are also summarized in Table 1.

**Table 1:** Key components of OBOT

Component	Details
1. Clinician(s) who prescribe(s) buprenorphine	Physician or other prescriber authorized to prescribe buprenorphine—until April 2021, limited to those who completed DEA waiver training
2. Nurses/other clinicians who support or lead care management	In most (but not all) models, the prescriber is supported by nurses and/or other clinicians who coordinate patient follow-up, buprenorphine prescription refills, drug testing, etc
3. Technical assistance	May include both training sessions and provider-to-provider consultations
4. Linkage to a ‘hub’	Optional: some OBOT clinics are linked to a ‘hub’ (typically an Opioid Treatment Program) where patients typically receive induction and stabilization, before transferring to the clinic (‘spoke’) for maintenance

### Division of labor

Among provider practices that offer OBOT, some have the physician conduct visits without staff assistance, while others designate a clinic staff member (often a nurse or social worker) to coordinate patient follow-up, buprenorphine prescription refills, drug testing, and prescription drug monitoring program queries as well as other functions [34]. In some models a nurse care manager conducts most of the visits, interspersed with less frequent physician visits. Coordinated care management is viewed as an efficient way for the prescribing provider to manage more patients [35], and can also facilitate connections for patients who need medical care or additional mental health services, such as counseling [27].

### Technical assistance

An additional important component is the participation in technical assistance by practices adopting OBOT, including both training sessions and provider-to-provider consultations. In several states, the technical assistance has been delivered using adaptations of Project ECHO (Extension for Community Healthcare Outcomes) [36], and the Providers Clinical Support System [37].

### Delegation of induction/stabilization

In a number of states, OBOT clinics can affiliate with a ‘hub and spoke’ network. As originally designed in Vermont, this approach creates ‘hubs’, typically at OTPs, for patients with more severe OUD, and ‘spokes’, which are primary care practices that offer OBOT for more stable cases of OUD as well as delivering psychosocial services [38, 39]. Patients typically receive induction and stabilization at the hub, and are then referred back to the primary care spoke for maintenance, and return to the hub if they destabilize. The hub and spoke model has also been adopted and in some cases modified elsewhere, including in Washington state [40] and California [41], sometimes using sites other than OTPs as hubs. In Vermont**, s**pokes must be staffed by at least one nurse and one mental health counselor per 100 patients [42]. At each spoke, care is coordinated by a registered nurse clinician case manager and/or care connector (peer or behavioral health specialist). In addition, hub clinicians provide consultative services to the spokes, and are available to manage clinically complex patients or support tapering of MOUD [34].

A number of different models have emerged for delivery of OBOT, which differ in how they organize the above-listed components. Further details are provided in a recent report to the Agency for Healthcare Quality and Research [35].

## Economic framework

For a clinic considering adoption of OBOT, there are investment costs involved, such as spending on recruiting staff, adapting computer systems, etc., and these can present a barrier. However, we start out by focusing instead on how OBOT adoption might affect operating margins, using a budget impact analysis. Consider an organization that is reimbursed on a fee-for-service basis. Suppose the organization delivers only two types of service: one type is billable (b) and the other is nonbillable (n). Its operating margin can be written as follows:

$${\text{Margin}}\, = \,{\text{Total}}\,{\text{revenue}} - {\text{total cost}}\, = \,{\text{f}}_{{{\text{bqb}}}} + {\text{G}} - {\text{C}}\left( {{\text{qb}},{\text{qn}}} \right).$$

Where f_b_ is the fee for a billable service; q_b_ and q_n_ are the volumes of billable and nonbillable services respectively; and G is any ‘external’ funding not linked to volume of service (e.g., grants). The organization’s total revenue depends mainly on the volume and fee paid for billable services, whereas its total cost (C) depends on the volume of both billable and nonbillable services (and how those volumes affect unit cost, which may include effects of one service on the cost of another). Of course, the reality is typically more complicated than this, as most organizations deliver multiple types of billable (and nonbillable) service, with fees and costs that may differ for each type. But this equation highlights the importance of several issues to look for when considering OBOT adoption:

- Impact on the volume of non-billable services (q_n_). Typically, this will increase, adding to the organization’s total cost. But how large an increase can be expected?
- Impact on the volume of billable services (q_b_). Will OBOT lead to more use of services for which the clinic’s parent organization can bill payers, such as pharmacy? This will increase cost, but also revenue, so another question is: how large is the organization’s margin (fee minus unit cost) on those additional billable services?
- Impact on the cost of billable services (C). Will OBOT (and delivery of more nonbillable services) reduce the cost of delivering certain billable services? For example, use of a (nonbillable) nurse care manager could reduce the provider’s paperwork burden before and after visits, thus reducing the cost per visit through task-shifting. Similarly, the more billable visits each salaried OBOT provider delivers (higher productivity), the lower the unit cost of each visit.

If this initial analysis indicates that the clinic’s operating margin is improved by adopting OBOT, one can proceed to a return-on-investment analysis, and ask how long a timeframe will be required to recover the initial investment, and how it could be funded. Conversely, if this analysis indicates a worsening of the clinic’s operating margin, the investment would not be recoverable. From the clinic’s perspective, further analysis is presumably unnecessary, even though there might still be net benefits from a societal perspective.

## Funding the new services needed for OBOT

From the above, it may be seen that the critical challenge for clinics is how to fund the new types of services involved in OBOT. Many clinics receive much of their funding from Medicaid, Medicare and private payers, and are paid largely on a fee-for-service basis, even if a managed care plan pays them. Under fee-for-service reimbursement, providers can typically bill for the medication itself (under a plan’s pharmacy benefit), and for the associated physician visit. However, some of the novel services may not be billable. Most prominently, this applies to the nurse care manager position, but it is also relevant to services like administrative support, and specialist training or consultation to primary care providers, which are typically not billable under FFS because there is no doctor-patient interaction [35]. In this section we outline several funding models, and their implications for clinics. Which options are available mostly depends on the rules set by states and insurers, and is thus usually beyond the control of the individual clinic.

### Billability of the novel services

For a clinic considering adopting OBOT, the best scenario (but not the usual one) is when it can actually bill for the novel services involved. For example, in Massachusetts, community health centers that are federally qualified (FQHCs) are able to bill for NCM visits at a comparable rate as for other licensed clinical providers. This opportunity creates the potential to support the NCM salary out of fee-for-service billing of OBOT services [43]. The minimum caseload needed to achieve this is further discussed below.

A more limited version of this ‘billability’ occurs at hospital-based clinics which are allowed to charge a facility fee for nurse services, even though the professional services are not billable.

### Cross-subsidization from higher fees for billable activity

If a payer enhances fees for billable services, the clinic can use that extra revenue to support its non-billable activities (cross-subsidization). In 2017, Virginia’s state Medicaid program started offering waivered office-based providers enhanced fees for MOUD treatment and care coordination services, as well as exemption from prior authorization requirements for those services. These benefits are available to waivered clinicians who apply to become “preferred OBOT” providers, as long as their site also has behavioral health clinicians [44]. By 2020 the state had more than 100 preferred OBOT providers. One could interpret the higher fees as a cross-subsidy to help the clinic to cover its cost of non-billable services, not unlike the grant funding used in Massachusetts. However, Virginia also established a monthly bundled rate ($243) specifically for SUD-specific care coordination, a more direct subsidy to nonbillable services (as discussed more in Sect. “Funding from alternative payment models”).

### Cross-subsidization from profits on additional billable activity

A third possible model is to fund the non-billable services using the margins obtained on additional billable activity, assuming that these are positive. Positive margins depend on productivity being high enough to keep the unit cost per OBOT visit below the reimbursement rate. In several of the case studies reviewed below, financial viability depended on assumptions about staff productivity. For example, in a simulation study Fried et al. used assumptions about physician hours and NCM hours provided per patient per year in each of 4 delivery models (e.g., 2.0 and 4.0 respectively in a nurse-led model)[45].

Similarly, in one health system in western North Carolina, Farrar et al. [46] report that adoption of OBOT resulted in a net increase of 1.93 medical visits per patient per month among OUD patients. Under a series of assumptions reviewed below, they concluded that treating 1 existing patient for 1 year would generate $1439, and treating 1 new patient would generate $1677. Across multiple patients, the additional revenues could be sufficient to support a NCM position.

This funding model is better suited to larger clinics, which can generate enough additional revenue to fund the new services. It is also better suited to more integrated health care systems, which may be able to capture additional revenues generated for services such as pharmacy, in addition to the OBOT revenue, to offset their costs of delivering nonbillable services. For example, the setting studied by Farrar et al. was a community-based family medicine residency clinic with integrated behavioral medicine and clinical pharmacy. More generally, OBOT may generate additional pharmacy billing, but that revenue will not be captured by a clinic that has no pharmacy (as in many traditional primary care clinics), or a clinic that has one but most patients fill their prescriptions elsewhere. Similar considerations apply to other types of revenue generated outside the clinic. If the clinic is part of a hospital system, the question becomes whether system administrators recognize the additional revenue that the OBOT clinic is generating for it, making them more willing to sustain that investment. Without that recognition, system administrators will likely view the OBOT clinic purely as a cost center and be correspondingly less willing to invest. For example, one clinic computed how many patients used the hospital’s pharmacy on the same day they had an OBOT visit, as a measure indicating possible revenue gains from OBOT.

An additional concern would apply if some of the additional visits generated are delivered via telemedicine, as is common during the current Covid-19 pandemic. Some insurers pay less for telemedicine than for in-person visits, which would reduce the revenue gained from any extra visits.

### External funding of nonbillable services

Some states fund nonbillable OBOT services through grants they receive from SAMHSA under the State Targeted Response (STR) and State Opioid Response (SOR) programs. Another growing resource for states are Section 1115 demonstration Medicaid waivers of Institution for Mental Disorder (IMD) exclusion from the Centers for Medicare and Medicaid Services, which are intended to improve care for substance use disorder (SUD), serious mental illness and severe emotional disturbance[47]. With these waivers, CMS allows states to bill Medicaid for care in previously ineligible inpatient and residential settings, enabling states to use some of the funding thus freed up from the federal match to expand community-based treatment. CMS has made state investment in evidence-based care for SUD and co-occurring disorders, including expansion of MOUD, a condition of obtaining such waivers. So far, 32 states have Medicaid 1115 waivers for IMD exclusion for SUD treatment approved, and 3 more have waivers pending [48].

Some health centers have been able to tap external (non-fee-for-service) funding to pay for nonbillable services, such as the NCM staff position. In Massachusetts, the state-initiated rollout of OBOT at community health centers included state block grants to cover nonbillable services. These included the salary of a full-time registered nurse, staff training, and technical support on implementation of protocols for each program. More recently the state switched to funding those nonbillable services with a ‘unit rate’. In most other states, clinics do not have access to such funding. However, some clinics have been able to access funds from the Health Resources and Services Administration (HRSA). Similarly in Vermont, for every 100 Medicaid patients receiving MOUD at a ‘spoke’ (OBOT) practice, the state Department of Health Access pays the cost of one nurse and one licensed mental health/addiction counselor to support the prescribing providers [49].

Expert consultation to OBOT providers, being similarly nonbillable, has also been implemented using grant funding from SAMHSA to states (as in Massachusetts and California [50]), or from universities and foundations (e.g., in North Carolina [51] and West Virginia [52]). In Massachusetts, a network of expert faculty is paid a retainer by the Medicaid carve-out to take calls from primary care providers regarding MOUD and other opioid prescribing. Again, this is supported from Medicaid 1115 waiver funding.

### Funding from alternative payment models

Another approach to funding OBOT would be for payers to provide a preset amount to spend on treating OUD, which is not conditioned on the number of specific services provided. This approach is most often used by managed care payers.

One example of this type of ‘alternative payment model’ is bundled payment (a preset amount per treatment episode). Bundled payment is used by some state Medicaid programs (e.g., Rhode Island) to reimburse OBOT [34], and similar payment models for OBOT have been developed by the American Society for Addiction Medicine [53], and by the Innovation Accelerator Project at the Centers for Medicare and Medicaid Services (CMS) [54]. A major advance occurred in January 2020, when Medicare introduced a new monthly bundled payment option for OBOT, supported by three new procedure billing codes. Code G2086 covers “office-based treatment for OUD, including development of a treatment plan, care coordination, individual therapy and group therapy and counseling; at least 70 min in the first calendar month.” Code G2087 covers at least 60 min in a subsequent calendar month, for the same services (except for development of a treatment plan). Code G2088 covers the same services as G2087 for each additional 30 min beyond the first 120 min. Notably, these codes do not cover payment for the OUD medications or for related toxicology testing, which continue to be billed separately. The codes are also not usable for patients concurrently seen at an OTP, as CMS set up a separate bundled payment rate for OTPs. Interestingly, the codes are also usable for visits delivered as telemedicine. Responding to earlier comments on its proposed rule, CMS said it would consider later refining the codes to adjust for different levels of patient need [55]. In addition, various states are reportedly considering applying a Medicare-type bundled payment approach in their Medicaid programs.

Another alternative payment model is capitation, where the provider is paid a preset amount per plan member per month, regardless of actual service use. Several states (including Maine, Maryland, and Vermont) have used funding from Medicaid’s health home program to encourage providers to set up ‘opioid health homes’ paid on a capitated basis, as a way to cover non-billable services such as care management and care coordination. Maine’s Medicaid program pays opioid health homes capitation amounts that range from around $400 to over $2200 per member per month, depending on the level of care a patient needs and whether the health home also provides coordinated case management [56]. A key component of this model is who is identified as a health home member, with some states (e.g., Maine) asking patients with OUD to opt in, while others auto-enroll them with an option to opt out [56].

If a clinic’s only revenue source is capitation, the distinction between billable and non-billable services disappears, so the clinic has greater flexibility in its staffing decisions. Novel services like NCM are then on an equal footing with traditional services, and the clinic’s decision can focus instead on whether or not hiring an NCM will reduce its other costs. And similarly for physician consultation and other services that were non-billable under a fee-for-service model. Of course, if some payers continue to pay fee-for-service, the clinic cannot so readily abandon the billable/non-billable distinction. Capitation offers the provider greater flexibility than bundled payment.

From the clinic’s perspective, a key question about funding under alternative-payment models is the adequacy of the preset payment amount, and what level of service provision it assumes. For example, in setting the payment rates for its Medicare OBOT bundled payments, CMS assumed an average of two individual psychotherapy sessions per month and four group therapy sessions per month, while recognizing there will be variability from patient to patient and over time. Similarly, state Medicaid programs use prospective (daily or monthly) rates to pay both FQHCs and CCBHCs for OBOT, and the rates are reportedly sometimes more generous for CCBHCs. In the case of capitation, revenue is tied to enrollment, and clinics face lost revenue on patients who are periodically disenrolled from Medicaid.

## Evidence on requirements for financial sustainability

Next, we review some economic analyses of OBOT sustainability conducted for existing programs in Massachusetts, North Carolina and Minnesota, and in a simulation using data from 20 clinics nationwide. These examples were identified as being ones which provided adequate information about their methods and assumptions. Their characteristics are summarized in Table 2.

**Table 2:** Table comparing case studies of OBOT

Example study	Massachusetts CHC [43]	North Carolina [46]	Minnesota	National simulation [45]
Type of setting	Community health centers	Primary care system	Safety-net hospital	CHC, other clinics
OBOT design features				
1. Clinician type that leads and bills induction visits	Nurse care manager	Nurse practitioner or clinical pharmacist	Physician, nurse practitioner, or physician assistant	Varies across models
2. Clinician type that manages care	Nurse care manager		Physician, nurse practitioner, or physician assistant	Varies across models
3. Technical assistance to OBOT team	Day-long training plus ongoing support	Not specified	Addiction specialty team for day-long training and academic detailing; ECHO community	Not specified
4. Clinic linked to a ‘hub’?	N	N	Y	N
Financing				
1. Nurse visits billable?	Y	N	Facility fee only	Y
2. Enhanced fees for preferred OBOT providers	N	N	N	N
3. Cross-subsidization from profits on new billable activity	Y	Y	Y	Y
4. Use of grant funding	Y	N	N	N

### Massachusetts FQHC experience

The ‘Massachusetts model’ was developed in order to expand Boston Medical Center’s OBOT Collaborative Care Model to community health centers (CHCs), from its initial base at an academic medical center. The model involves hiring nurse care managers (NCMs) at each CHC to provide waivered providers with clinical support to manage patients with OUD on buprenorphine [43]. Among other roles, the NCM (a) performs the initial screening, for patients seeking OBOT treatment, after which the patient sees the waivered provider; (b) assesses the patient for withdrawal symptoms following a protocol; and (c) supports the patient through the induction process under the orders of the waivered provider. Initially, patients are required to see the NCM weekly for follow-up visits with drug screening and verification of behavioral health counseling, although this frequency can decrease depending on progress [43].

An analysis of the Massachusetts FQHC OBOT program concluded that having a NCM provide OBOT is sustainable and viable in a Federally Qualified Community Health Center, at least in that state [43]. The analysis found that it would take approximately 40 cases per year, at 27 visits per patient per year to fund a full-time NCM position, after adjusting for efficiency and administrative cost. A typical NCM’s caseload was initially 100 patients, more than twice the number of cases required [43]. The results depend on assuming 90% utilization for a caseload with varying frequency of visits. The authors noted that even in Massachusetts, not all CHCs could use that funding model, because only FQHCs were paid prospectively by Medicaid and were able to bill individual medical visits delivered by nurses at the same rate as those delivered by physicians [43]. Thus, this analysis would not apply in the many states and contexts where NCM services are non-billable.

A later report noted that 7 of the Massachusetts FQHCs were able to expand beyond grant funding and add an additional NCM, which appeared to support the earlier conclusions about financial sustainability [57].

### North Carolina health system experience

In the North Carolina study, Farrar et al. concluded that the additional billable revenue generated by delivery of OBOT had created positions for 2 licensed clinical addiction specialists and 1 peer support specialist [46]. They estimated that delivering OBOT to 100 existing OUD patients increased the clinic’s revenue by $143,861, through increased delivery of billable medical visits. For 200 patients, the revenue would be double that amount. Their model included assumptions that (a) all visits were reimbursed using the North Carolina Medicaid Physician Fee Schedule (presumably lower rates than other payers); and (b) the costs to be covered included 20% ‘cost of business’, e.g., central business office, facilities etc. A weakness of the design was lack of a control group, implying that some of the increase in visits that they observed could have resulted from influences other than delivery of OBOT, e.g., the patients’ course of disease, or policy changes besides OBOT [46].

### Minnesota safety-net hospital experience

In 2017, an OBAT program was launched at Hennepin County Medical Center, the largest safety-net hospital in Minnesota, led by one of the authors (GB). This clinic is mostly paid fee-for-service, most patients are uninsured or publicly insured, and NCM services are non-billable, although payers reimburse a facility fee for nurse services provided in hospital-based ambulatory care clinics. In the planning stages, medical center staff analyzed the financial impact of investment in an OBOT clinic. They projected that in the first year, operating surplus would not cover the overheads, but thereafter it would exceed overhead, resulting in a net surplus. This improvement did occur. In the model, financial improvement resulted from an assumed growth in physician productivity over time, as the program ramped up. (Beverlee Shellum, personal communication).

Key assumptions in this Minnesota modeling included the number of annual visits each physician could provide (840 for new patients and 2100, later 5040, for established patients); how many annual visits each nurse could provide (1932); the facility fees for physician and nurse services; the percent of billed charges actually collected (37.5%); and the overhead charged by the parent entity (about 8% on top of actual expenses). Various of these assumptions could be adapted by other organizations considering OBOT adoption, if they conduct similar analyses.

### *Fried *et al*. simulation*

Fried et al. recently published a study simulating the effect of OBOT adoption that was based on the experience of 20 primary care practice leaders engaged in buprenorphine prescribing in the United States [45]. They considered the effects of 4 model types (with varying division of labor among clinicians) in 4 different settings (varying by rurality, poverty and whether a CHC). For many parameters they used averages of values supplied by the study respondents.

Fried et al. concluded that any of the 4 approaches they considered would be expected to show an overall increase in net patient revenue, relative to the status quo of referring patients to other providers for addiction care. However, providers would need to maintain at least 9 patients in treatment and no-show rates of < 34%. The savings are expected to be larger with a nurse-care manager model than with physician-led approaches. This is probably because the NCM model achieves higher productivity, reducing the unit cost per visit relative to the reimbursement rate.

### Summary of case studies

The preceding case studies can also be classified according to the various funding approaches presented in Sect. “Funding the new services needed for OBOT”. The Massachusetts example used grant funding and the billability of NCM visits. Lacking that advantage, the North Carolina and Minnesota clinics appear to have succeeded by generating additional billable services at a low enough unit cost to allow them to cross-subsidize nonbillable services. In the Fried study, models with more reliance on nurse care had more billable services to cover, but also looked more financially viable. The Minnesota clinic was the only one of these examples affiliated with a hub-and-spoke model (it was a hub).

## Discussion

The evidence reviewed in this paper indicates that there is considerable variation in the reimbursement environment facing clinics that are considering adopting OBOT, including a variety of ways to cover the cost of the nonbillable activities required. Some clinics may have access to state or federal grants to support nonbillable activities, although often those grants are time-limited, posing additional challenges for sustainability. Alternatively, clinics may be able to capture revenues from extra services generated by OBOT adoption, and from keeping unit costs on billable services below the reimbursement rates. Other clinics may not have those opportunities, depending on their organizational affiliations and the policies of the state where they operate. The new Medicare bundled payment is a potential game-changer, given the program’s size and its historic reliance on fee-for-service payment, and it could be helpful for clinics where a substantial proportion of OUD patients are covered by Medicare. Small clinics reimbursed mostly fee-for-service may be particularly challenged in pursuing OBOT, given the need to fund a dedicated staff and extra administrative work.

Clinics considering adoption of OBOT are well advised to thoroughly investigate these issues as they make their decision. Table 3 provides some examples of questions to ask, and Table 4 lists some of the key parameters identified in the case studies. It will be important for these conversations to include administrators familiar with billing implications as well as clinicians who understand the delivery models. Some online resources exist for clinics that are considering OBOT adoption [58], although given the regulatory differences across states, a state-specific resource is most likely to be useful.

**Table 3:** Questions for a clinic considering OBOT adoption

1. If your organization is primarily paid on a fee-for-service basis
a. Will payers increase your billable rates to cross-subsidize non-billable OBOT services?
b. Will your OBOT service volume be sufficient to provide revenue that could support the salary of staff delivering non-billable services?
c. Will OBOT adoption result in additional volume of other billable services (e.g. pharmacy) that could cross-subsidize non-billable OBOT services?
d. Are other subsidies available to support non-billable OBOT services? (e.g. grants from the state or from HRSA)
2. If your organization is not primarily paid on a fee-for-service basis
a. Are your non-fee-for-service payment rates (e.g. bundled payments) adequate to pay for all needed OBOT services?
b. Are other subsidies available to supplement the payment rates? (e.g. grants from the state or from HRSA)

Evaluation: if your answers are mostly ‘no’, then OBOT implementation will be more challenging for your clinic

**Table 4:** Key parameters that may the affect financial viability of an OBOT program

Parameter
Productivity of OBOT team (visits per FTE)
Reimbursement rate for OBOT visits, by provider type
Facility fee for OBOT visits, by provider type
Average reimbursement rate for other services (opportunity cost)
Prevalence of opioid use disorder
Proportion of patients accepting therapy when offered
No-show rate for OBOT visits
Panel size, all patients per full-time physician
Percent of patients Enrolled With Medicare, Medicaid, Private, Uninsured
Salary per year for Nurse care manager; behaviorist
Overhead rate

Note: Many of these parameter values would depend on the staffing model selected

In turn, this wide variation in payment approaches has implications for researchers and policymakers. For researchers, the variation offers the opportunity to compare the impact of different payment approaches on the speed with which OBOT diffuses, as well as downstream effects on patient outcomes. Research on adoption and financial sustainability of OBOT will need to consider costs and revenues from the provider viewpoint, in addition to any consideration of societal cost-effectiveness. Since the evidence we present for the different financial strategies mostly derives from case studies, it would be helpful to have more studies that cover a larger number and variety of settings (as Fried et al. attempted), in order to identify whether and how different strategies may promote adoption of OBOT, or allow it to reach more patients once adopted. It would also be valuable to conduct more research (including qualitative work) on providers' perspectives, to understand which financial models would make OBOT adoption more attractive to providers.

For policymakers, the motivation to commission and use such research should be strong, given the apparent role of financial barriers in impeding the uptake of OBOT. Better information would allow them to adopt policies that are more successful in making OBOT more widely available, and increase the numbers in treatment. In particular, if approaches such as bundled payment do not fully address the technical assistance and provider education needs required to scale up OBOT, then state and federal policy makers may need to provide alternative funding streams for those needs.

More generally, it is worth noting that many of the reimbursement challenges in this paper are by no means unique to OBOT. Other novel treatment models have faced similar problems due to their reliance on services which are ‘non-billable’ in a fee-for-service environment. These other models have included screening and brief intervention and referral to treatment (SBIRT) for substance use [59], and collaborative care for depression [60]. Some novel services can be made billable if insurers create new procedure codes (as has occurred with SBIRT and now OBOT), but achieving this is often a protracted process. Other novel services can be more difficult to fit into the fee-for-service model because they do not involve direct clinician-patient contact (e.g., consultation among physicians, health education, technical assistance to programs). This highlights the ways in which fee-for-service reimbursement is challenging for integrated care models more generally, which is one reason why many payers are increasingly trying to reduce their use of fee-for-service reimbursement in health care.

The current pandemic may pose both opportunities and challenges for the sustainability of OBOT. On the one hand, the Drug Enforcement Administration recently started allowing teleprescribing of buprenorphine [61], and multiple insurers have started temporarily reimbursing OBOT delivered through telemedicine, which was previously not covered. These changes could expand the reach of OBOT [62]. On the other hand, some of the insurers covering telemedicine are paying at lower rates than for in-person visits, meaning a revenue loss for clinics which have to deliver some of their OBOT remotely. There is also uncertainty about what will happen to the recently implemented pandemic-related policies which have improved access to OBOT, after the pandemic abates.

## Conclusion

The reimbursement environment for OBOT delivery varies widely around the US, and is evolving as Medicare, Medicaid (and possibly other payers) introduce alternative payment approaches. Clinics considering adoption of OBOT are well advised to thoroughly investigate these issues as they make their decision. In addition, payers will need to rethink how they pay for OBOT in order to make it financially sustainable.

## Data Availability

No datasets were created in the development of this article.

## Abbreviations

CHC: Community health center
CMS: Centers for Medicare and Medicaid Services
DEA: Drug Enforcement Administration
FFS: Fee-for-service
FQHC: Federally qualified health center
MOUD: Medication for Opioid Use Disorder
NCM: Nurse care manager
OBAT: Office-Based Addiction Treatment
OBOT: Office-Based Opioid Treatment
OTP: Opioid treatment program
OUD: Opioid use disorder
